# The performance of silicon photomultipliers in Cherenkov TOF PET

**DOI:** 10.1186/2197-7364-2-S1-A1

**Published:** 2015-05-18

**Authors:** Rok Dolenec, Samo Korpar, Peter Krizan, Rok Pestotink

**Affiliations:** Faculty of Chemistry and Chemical Engineering, University of Maribor, Maribor, Slovenia; Jozef Stefan Institute, Ljubljana, Slovenia

In time-of-flight positron emission tomography (TOF PET) one of the main factors limiting the time resolution is the time evolution of the scintillation process. This can be avoided by using exclusively the Cherenkov light produced in a suitable material. Sub 100 ps FWHM timing has already been experimentally demonstrated but with a drawback of relatively low detection efficiency due to the photodetectors used. In this work silicon photomultipliers (SiPMs) are considered as a photodetector in Cherenkov TOF PET. The detection efficiency can be significantly improved by using SiPMs, however, at room temperature the SiPM dark counts introduce a significant source of fake coincidences. SiPM samples from different producers were tested in a simple back-to-back setup in combination with lead fluoride Cherenkov radiators. Results for coincidence timing, detection efficiency and effects of dark counts at different temperatures and SiPM overvoltages are presented.

